# Mechanical ventilation in the ICU- is there a gap between the time available and time used for nurse-led weaning?

**DOI:** 10.1186/1757-7241-16-17

**Published:** 2008-12-02

**Authors:** Britt Sætre Hansen, Wenche Torunn Mathiesen Fjælberg, Odd Bjarte Nilsen, Hans Morten Lossius, Eldar Søreide

**Affiliations:** 1 Departments of Anaesthesia and Intensive Care, Stavanger University Hospital, Stavaner, Norway; 2 Faculty of Social Sciences, University of Stavanger, Stavanger, Norway; 3 Norwegian Centre for Movement Disorder, Stavanger University Hospital, Stavanger, Norway; 4 Department of Mathematics and Natural Science, University of Stavanger, Stavanger, Norway; 5 Department of Research and Development, Norwegian Air Ambulance, Drøbak, Norway

## Abstract

**Background:**

Mechanical ventilation (MV) is a key component in the care of critically ill and injured patients. Weaning from MV constitutes a major challenge in intensive care units (ICUs). Any delay in weaning may increase the number of complications and leads to greater expense. Nurse-led, protocol-directed weaning has become popular, but it remains underused. The aim of this study was to identify and quantify discrepancies between the time available for weaning and time actually used for weaning. Further, we also wished to analyse patient and systemic factors associated with weaning activity.

**Methods:**

This retrospective study was performed in a 12-bed general ICU at a university hospital. Weaning data were collected from 68 adult patients on MV and recorded in terms of ventilator-shifts. One ventilator-shift was defined as an 8-hour nursing shift for one MV patient.

**Results:**

Of the 2000 ventilator-shifts analysed, 572 ventilator-shifts were available for weaning. We found that only 46% of the ventilator shifts available for weaning were actually used for weaning. While physician prescription of weaning was associated with increased weaning activity (p < 0.001), a large amount (22%) of weaning took place without physician prescription. Both increased nursing workload and night shifts were associated with reduced weaning activity. During the study period there was a significant increase in performed weaning, both when prescribed or not (p < 0.001).

**Conclusion:**

Our study identified a significant gap between the time available and time actually used for weaning. While various patient and systemic factors were linked to weaning activity, the most important factor in our study was whether the intensive care nurses made use of the time available for weaning.

## Background

Mechanical ventilation (MV) is a key component in the care of critically ill and injured patients. Almost half the time patients spend on mechanical ventilation is devoted to weaning [1]. Delays in weaning the patient from MV increase the number of complications and may lead to increased expenditure [2]. Consequently, weaning constitutes a major challenge for the intensive care staff. It is important to wean the patient from MV as expeditiously as possible. Several studies [3-6] indicate that the implementation of nurse-led, protocol-directed weaning reduces the amount of time spent on MV, the length of ICU stay, and associated costs.

The introduction of nurse-led weaning under a protocol constitutes a systematic approach to weaning with less freedom for the individual clinician to decide if and how weaning should be performed [1,7]. This approach also facilitate teamwork and interprofessional communication and may therefore increase the success of weaning [8]. On the other hand, there are significant barriers to the use of such standardised evidence-based treatment protocols. For example, providers may be unaware of their existence, there may be a lack of agreement between physicians, or the providers may be unable to implement the protocols [9]. Alm-Kruse et al. [10] noted that involving nurses in the implementation of new therapies resulted in commitment, confidence and a "sense of ownership" that improved performance.

Weaning criteria have been widely discussed, and there now seems to be some international consensus on the matter[11]. However, there has been less focus on the process itself. For example, few measures have been reported of how available weaning time is actually used at the bedside and which factors that may be associated with weaning activity.

Similar to the majority of other Norwegian ICUs, we participated in the national ICU "Breakthrough" project in 1999 that focused on improving weaning from MV [12]. Unlike results reported in Brattebø et al. [12], our facility did not observe experience a reduction in the duration on ventilator (DOV) time as a result of this project. More knowledge of the organisational aspects of weaning seems to be warranted in order to improve weaning. Therefore, the aims of this study were 1) to identify possible discrepancies between the time used for weaning and time available for weaning and 2) to analyse the patient and systemic factors were associated with the time available for weaning that is actually used for weaning. To the best of our knowledge, these topics have not been studied to date.

## Methods

This study is a part of a larger initiative that aims to identify intensive care nurses (ICNs) and ICU physician perceptions of nurse-led weaning as well as aspects that are believed to encourage interprofessional collaboration in the weaning process. Qualitative (focus-groups) methods have also been used [13,14]. To determine if a selection of system and patient factors (independent variables) were associated with whether the time available for weaning (defined as weaning shifts which are 8-hours day-evening- and night nursing-shifts) was used for this purpose (the dependant variable), we performed a multivariate analysis using logistic regression (SPSS, version 15). Pearson's chi-squared test was used to test for differences in proportions across categorical variables and Mann-Whitney U test for continuous variables. Two-sided p-values less than 5% were considered statistically significant [15].

### Clinical setting

**Figure 1:**
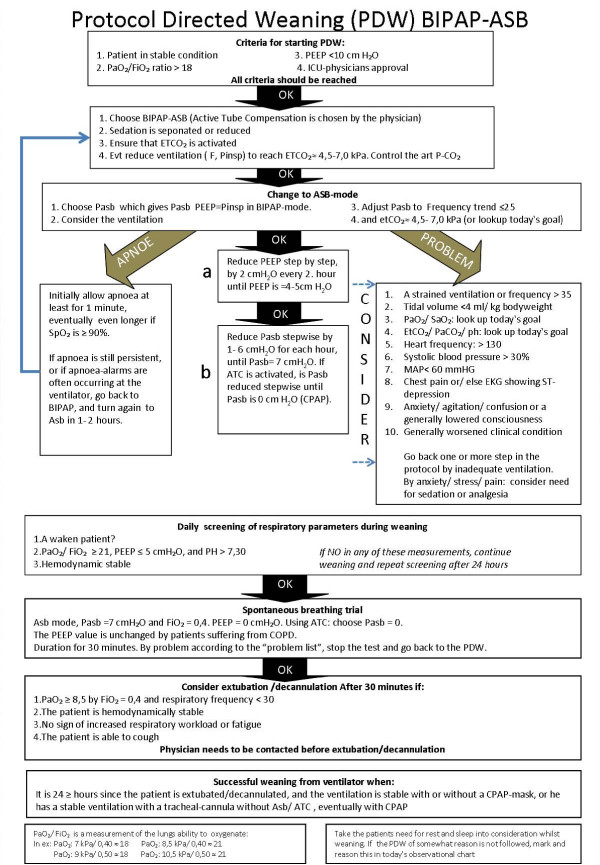
Weaning protocol (Appendix).

This retrospective study was performed in a 12-bed general intensive care unit (ICU) at a 700-bed University Hospital in Stavanger, Norway. Except for neonates, this ICU treats all patients with a need for MV in the hospital. It is a closed unit run by the Departments of Anaesthesia and Intensive Care. Anaesthesiologists work as ICU physicians. The daytime medical staff consists of two senior ICU physicians (including the medical director) as well as 1–2 anaesthesiology residents rotating through the intensive care service. One anaesthesiology consultant or senior resident covers the night shift. The ICU physician in charge is expected to determine daily goals for each patient, including the PDW (Figure 1) and level of sedation. The ICU physicians can use a modified weaning plan at their discretion. In March of every year, all ICNs are certified/re-certified in the various aspects of mechanical ventilation (including the use of the weaning protocol).

The ICNs rotate between the ICU and Postoperative Recovery Unit. A total of 125 nurses including managers and assistant nurses, share 88 positions in the Department of Intensive Care. We use the Dräger ventilator (Evita 4 and XL) and aim for a 1:1 nurse-patient ratio. The PDW includes a daily spontaneous breathing trial (SBT) [16,17] and weaning is initiated according to the four criteria listed in the PDW (Figure 1). Our sedation protocol is based on the use of midazolam and morphine, but it allows for propofol/fentanyl as well. The ICU physician and ICN decide on the preferred level of sedation, which is measured based on the Motor Activity Assessment Scale (MAAS) [18], as well as whether to use a bolus or continuous infusion for sedation. The importance of keeping the patient awake as much as possible during daylight hours is highlighted in the sedation guidelines.

### Patients and participants

Four experienced ICNs (including the first and second author) collected the data using written, predefined criteria for ventilator shifts and weaning activity (see below). All adult (16 years and older) patients undergoing more than 24 h of MV in our ICU during Oct-Nov in 2002, 2003 and 2004 were included. Patients with coincidental neurological disease were excluded. The data were collected in 2004–5 from daily ICU recording charts (from 2002, 2003, 2004), which are used by both ICNs and ICU physicians as a working tool. A total of 68 patients were studied (Table 1).

**Table 1:** Patient characteristics

		**N**	**(%)**	**Median**
Number of patients	Men	38	(56)	
	Women	30	(44)	
	**All**	**68**	**(100)**	
				
Age (median)	Men			63
	Women			59
	**All**			**63**
				
Diagnosis*	ARF alone	22	**(**32)	
	ARF plus trauma	15	**(**22)	
	ARF plus septic shock	12	**(**18)	
	ARF plus neuro-int.	19	**(**28)	
	**All**	**68**	**(100)**	
				
Diagnosis group	Medical patients	41	**(**60)	
	Surgical patients	27	**(**40)	
	**All**	**68**	**(100)**	

* ARF = mechanical ventilation for more than 24 hours.

### Ventilator periods and shifts

• *A ventilator period *is defined as the time from the start of mechanical ventilation until extubation, or reaching a minimum PEEP level of 5 cmH_2_O and patient-trigged, inspiratory pressure level of 7 cmH_2_O. If the patient was disconnected from the ventilator for more than 24 hours and then reconnected, we counted this as a new ventilator period.

• *A ventilator shift *is defined as an 8-hour shift (day, evening, and night shift) for one MV patient.

For each ventilator shift, we collected data regarding the following patient factors: age, diagnosis, acute respiratory failure (ARF) alone or with trauma, septic shock or neuro-intensive-problems, diagnosis group (surgical/medical), ventilator mode, NEMS [19], SAPS II [20], PEEP and tidal volume/kg (ventilator setting), drugs (sedation), FiO_2 _and heart rate. The following data on relevant systemic factors were also collected: year of data collection, time of day (day, evening, or night-shift), whether weaning was prescribed by physician and whether weaning efforts were performed according to the weaning protocol. The actual nurse:patient ratio and workload for each individual ventilator shift was not included as we found it impossible to collect precise data in a retrospective manner.

### Time used versus time available ratio

• A ventilator-shift used for weaning is defined as one nursing shift in which any alterations in the ventilator-settings were performed according to the weaning plan. Despite the fact that one alteration may not be considered sufficient to constitute a weaning effort, we chose this liberal definition to include all possible weaning attempts in our analysis.

• We define one ventilator-shift available for weaning based on the three criteria for physiological readiness to wean defined in the weaning-protocol (Figure 1). The forth criterion (weaning prescribed by a physician) was analysed as a systemic factor.

### Ethical considerations

We collected data from the ICU quality assurance database as well as ICU patient charts. The Norwegian Social Science Data Services approved (no. 11438) the data collection and storage of data. The Regional Ethical Committee regarded our study as a quality improvement study and declined to require informed consent from the patients.

## Results

**Figure 2:**
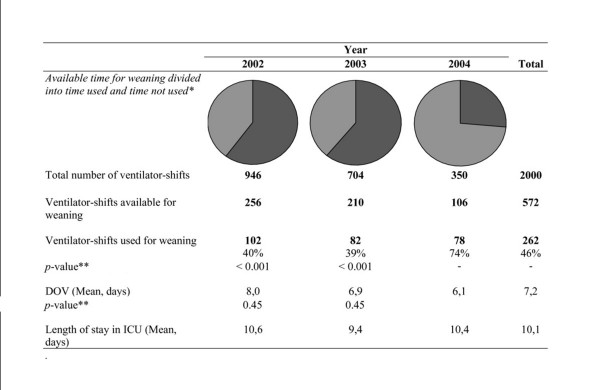
Available time for weaning divided into time used and time not used. DOV = duration of ventilation. *Light colour = Weaning, Dark colour = No weaning. ** 2002–2003 compared to 2004.

Data from the 68 patients (72 ventilator-periods) generated 2000 ventilator-shifts for analysis (Figure 2). Of the 572 ventilator-shifts available for weaning, 262 (46%) were actually used for weaning. In 2002 and 2003, roughly 40% of the available ventilator-shifts were used for weaning. This number increased to 74% in 2004 (Figure 2, p < 0.001). The significant increase in weaning activity was associated with an apparent reduction in the DOV (Figure 2).

We found a significant association between weaning prescription and weaning being performed (Table 2 and 3). However, in 127 (22%) of the available weaning-shifts weaning was performed without physician prescription (Table 2).

**Table 2:** The relationship between weaning prescribed and weaning being performed in the 572 available weaning shifts in the time period 2002 – 2004, p < 0.001.

	**Weaning performed**	**Weaning not performed**	**Total**
Weaning prescribed	135 (59%)	93 (41%)	228 (100%)
Weaning not prescribed	127 (37%)	217 (63%)	344 (100%)

Total	262	310	572

**Table 3:** Multivariate logistic regression of factors presumed associated with weaning.*

**Variable**	**Threshold**	**OR**	**95.0% CI for OR**		***p*-value**
				
	**Values**		**Lower**	**Upper**	
Year	=2004	7.21	3.79	13.74	< 0.001
Gender	=Man	0.88	0.59	1.32	0.544
Age	> 63	0.83	0.48	1.41	0.489
NEMS	> 34	0.95	0.91	0.99	0.012
SAPS		0.99	0.97	1.01	0.444
Peep (cmH_2_O)	> 5	0.63	0.42	0.93	0.020
Tracheostomy	=Yes	0.63	0.41	0.98	0.042
Prescribed by phys.	=Yes	2.60	1.78	3.80	< 0.001
Propofol	=Yes	1.35	0.84	2.19	0.218
TVKG (ml/kg)	> 7	1.17	0.79	1.73	0.444
FiO2	> 0.3	0.71	0.46	1.10	0.127
Heartrate	< 100	0.76	0.48	1.19	0.231
Systolic BP (mmHg)	< 150	0.92	0.57	1.48	0.723
Neuro_intensiv	=Yes	2.31	1.09	4.90	0.028
Night verus day/evening	=Night	0.37	0.24	0.56	< 0.001
Constant		25.33			< 0.001

*OR odds ratio, CI confidence interval.

Besides physician prescription, year of analysis (2004) and the presence of a neuro-intensive diagnosis were the only three factors significantly associated with weaning activity (Table 3). On the other hand, factors like increased workload (NEMS) and night shifts were associated with reduced weaning activity (Table 3).

Prescribed weaning did not increase during the period studied and remained around 40% (Table 2). However, there was a significant increase in weaning prescriptions that resulted in weaning efforts (46% in 2002 and 87% in 2004; p < 0.001). At the same time weaning during available shifts without physician prescription increased from 35% in 2002 to 63% in 2004 (*p *< 0.001).

## Discussion

The aims of this study were to define the time used versus time available for weaning and to study the patient and systemic factors associated with the available time actually used for weaning. We identified a significant discrepancy between the time used and time available for weaning. Because we used a liberal definition of weaning activity the results were quite surprising. This finding is in accordance with our previous studies [13,14], which showed that weaning frequently were given low priority despite being an essential part of the care of MV patients [11]. Therefore, we think measuring the time used versus time available for weaning can be a helpful way to demonstrate weaning status on an organisational level.

To better understand the under-use of the available weaning time, we analysed patient and systemic factors associated with the time available for weaning that was actually used for this process. Not surprisingly, we found that physician prescription was associated with more weaning activity and that night shifts, higher values for PEEP and NEMS were associated with less weaning activity.

Interestingly, a large amount of the weaning activity took place in ventilator-shifts without physician prescription. Further, there was an increase in weaning activity during the time period studied. We think these findings indicate that ICU physicians prescribe weaning too rarely. On the other hand when weaning prescriptions were issued, we found that the ICNs did not always follow them. Possible reasons for this failure to act may include the lack of continuity, lack of interprofessional collaboration and planning, lack of knowledge and experience and excessive workloads [13,14]. This conclusion is consistent with data reported by others [6]. ICNs may initiate weaning activity without prescription if physicians do not consider that prescribing weaning is their responsibility [13,14]. Regardless of whether weaning was prescribed, the ICNs tended to take an independent, leading role also suggested by Rose et al. [21]. Some ICNs took informal responsibility when the formal weaning procedure was not followed. This is an interesting finding that hospital decision-makers should be aware of.

Although our study design did not allow a full disclosure of all the mechanisms leading to improved weaning activity, we believe that the most likely explanation involves on-going educational efforts (certification and recertification). These efforts increased over the period studied, with maximum effort expended in 2004. Interestingly, these educational efforts may have resulted in improved weaning activity by the ICNs despite no increase in the number of weaning prescriptions issued by the ICU physicians. One explanation for this discrepancy may involve educational efforts concerning MV and weaning in our ICU, which were implemented separately and with different content for ICNs and ICU physicians. In processes like weaning that involve more than one group of caregivers interprofessional team-learning and reflection using shared mental models are important [8,22]. We therefore suggest that weaning outcomes should be discussed, reflected upon, reported, and measured on an interdisciplinary basis to motivate and stimulate the whole team. This method is in line with that proposed by Kassean and Jagoo [23] who recommend the creation of a climate that encourages open communication to overcome resistance to change.

The time used versus time available for weaning represents an analytical approach that may help us identify the causes of low weaning activity on both systemic and clinician levels. Based on such information, processes and practices to improve weaning activity can be discussed and implemented [13,14]. As ICU patients' situation and weaning readiness vary over time, teamwork and system-oriented thinking are crucial. Efforts and tasks that are not measured and reported on a regular basis are easily given low priority [13]. On the other hand, providing healthcare workers with feedback regarding weaning improvements in an easy and feasible manner can motivate and stimulate further improvements. As weaning activity may be fragmented and inconsistent due to the interest and level of knowledge of the individual healthcare workers, a collaborative and systematic approach is needed for success [24,25]. Implementing this novel "time used versus time available" ratio-approach to assess facility-wide levels of weaning activity may also help the individual clinician to identify his role in the weaning process.

Weaning protocols seem to be a good idea [26,8,3]. This study indicates, however, that neither protocols nor educational efforts will improve and facilitate weaning as a team process in the absence of systemic thinking. More interprofessional communication and planning may remedy this situation and streamline the process of weaning [7,9].

## Limitations

The present data come from a single ICU and may not necessarily be generalisable to other ICUs. Further, we used a very liberal definition of weaning despite our awareness of the validity and reliability problems involved. The criteria used to define the time available and time used for weaning were both based on our own protocol for weaning (see Figure 1), which again reflects existing international research on development of such protocols [2,4,16,17,27,28]. We used individual nursing shifts to provide an analytic context for weaning efforts over time. Both of these definitions may be criticised for representing a too mechanistic, static and simplified a view of the care giving framework and the "art of medicine". Further, we only included a limited number of patient and systemic factors in our multivariate analysis of the weaning activity. The nursing shifts studied were not independent observations, as the same patients may have contributed data to multiple nursing shifts. The significant increase in weaning activity was associated with an apparent reduction in the DOV from 8.0 in 2002 to 6.2 in 2004. The fact that this reduction did not reach statistical significance is more likely due to our limited sample size. Still, we suggest that our main finding, namely the large discrepancy between the time available and time used for weaning, exists and is valid. This discrepancy indicates that more studies on the organisational aspects of weaning are still needed.

## Conclusion

Our study revealed a large gap between the time available and time actually used for weaning. The time used versus time available for weaning ratio represents a new way of reporting the weaning status and process at an organisational level. Although various patient and systemic factors were linked to weaning activity, the most important factor in our study was whether the ICNs made use of the time available.

## List of abbreviations

PEEP: Positive end expiratory pressure; NEMS: the Nine Equivalents of nursing Manpower use Score; MAAS: Motor Activity Assessment Scale; SAPS: Simplified acute physiology score; ARF: Acute respiratory failure; DOV: Duration of ventilation; PDW: Protocol-directed weaning.

## Competing interests

The authors declare that they have no competing interests.

## Authors' contributions

BSH contributed to the data collection and was the primary author of the manuscript. WMF created the File Maker Pro database used to store our data, was in charge of the data collection, and contributed to authoring the manuscript. Both authors initiated the study. OBN translated the data into SPSS and generated the tables. HML contributed with valuable advice throughout the data collection period. ES facilitated the processes of data collection and writing.
